# 55 Initial Experience Using Artificial Intelligence for the Assessment of Pediatric Burn Depth

**DOI:** 10.1093/jbcr/irac012.058

**Published:** 2022-03-23

**Authors:** James H Holmes, Herb A Phelan, Jeffrey W Shupp, J Michael DiMaio, Jeffrey E Carter

**Affiliations:** Atrium Health Wake Forest Baptist, Winston-Salem, North Carolina; LSUHSC-New Orleans Department of Surgery, New Orleans, Louisiana; MedStar Washington Hospital Center, Washington DC, District of Columbia; Baylor Scott and White, Dallas, Texas; University Medical Center- New Orleans, New Orleans, Louisiana

## Abstract

**Introduction:**

Estimation of burn depth, and hence severity, is critical for burn management. Burn depth estimates vary widely, and these inaccuracies can be compounded in pediatric burns. A reliable, objective, non-invasive device for the accurate assessment of burn depth is needed. A non-invasive imaging technology, using multispectral imaging (MSI) combined with a machine learning algorithm (MLA), is being developed as a tool for burn depth assessment. The results of the initial multi-center study using this artificial intelligence (AI) technology in pediatric burns are presented.

**Methods:**

The MSI device was used to image subjects < 18y of age with thermal burns < 50% TBSA. It captured a set of images measuring the reflectance of visible and near-IR light, within a 23x23 cm field-of-view. Images were collected from up to 2 separate burned regions within 72 hours of injury that were then serially imaged for up to 7d post-injury. Burns that the investigator believed would heal spontaneously (superficial or superficial partial-thickness) were managed per institutional standard of care (SOC) and assessed at 21d post-injury for complete healing. Burns that the investigator felt would not heal by 21d post-injury (deep partial-thickness or full-thickness) were excised and grafted per institutional SOC, with multiple biopsies being taken prior to excision.

Regions of non-healing burn within every MSI image were identified by a panel of 3 burn surgeons. To accurately identify these non-healing regions, the panel of surgeons was given access to 1 of 2 clinical reference standards: a) the 21-day healing assessments for burns allowed to heal spontaneously; or b) pathology reports detailing histologic analyses from the biopsies.

This information was then used to develop a type of MLA called a convolutional neural network (CNN) that could automatically identify the regions of non-healing burn within an image. From these data, an ensemble of 8 separate CNN algorithms was used to automatically identify non-healing burn tissue. Training and test accuracies of the ensemble CNN were calculated using cross-validation at the level of the subject.

**Results:**

Twenty-four (24) pediatric burn patients were enrolled, with 26 burned areas being serially imaged. The age range of the subjects was 7 months - 17y, with a mean age of 5.7y. Subjects had a mean burn size of 8.0 ± 4.2% TBSA, and 70% of the subjects were male. The AI performance results showed an accuracy of 88.2 ± 3.7%, sensitivity of 80.0 ± 14.6%, specificity of 88.0% ± 3.7%, and an area under the curve (AUC) of 0.92.

**Conclusions:**

Our study demonstrates an improvement in the accuracy of burn depth assessment over the traditional exam, which could lead to improved burn care.

